# Standardised sporulation methods for *Diplodia*, *Lasiodiplodia* and *Neofusicoccum*

**DOI:** 10.3897/imafungus.17.176189

**Published:** 2026-02-06

**Authors:** David Hernández-Hernández, Felipe Siverio de la Rosa, Christiaan Grobler, Bernard Slippers

**Affiliations:** 1 Unidad de Protección Vegetal, Instituto Canario de Investigaciones Agrarias, 38270 Valle de Guerra, San Cristóbal de La Laguna, Canary Islands, Spain University of Pretoria Pretoria South Africa; 2 Escuela de Doctorado y Estudios de Posgrado, Universidad de La Laguna, 38200 San Cristóbal de La Laguna, Canary Islands, Spain Universidad de La Laguna San Cristóbal de La Laguna Spain; 3 Sección de Laboratorio de Sanidad Vegetal, Consejería de Agricultura, Ganadería, Pesca y Aguas del Gobierno de Canarias, 38270 Valle de Guerra, San Cristóbal de La Laguna, Canary Islands, Spain Unidad de Protección Vegetal San Cristóbal de La Laguna Spain; 4 Department of Biochemistry, Genetics and Microbiology, Forestry and Agricultural Biotechnology Institute (FABI), University of Pretoria, Pretoria 0028, South Africa Sección de Laboratorio de Sanidad Vegetal San Cristóbal de La Laguna Spain

**Keywords:** *
Botryosphaeriaceae
*, conidia, long-term storage, sporulation, Vogel’s Minimal Medium

## Abstract

Members of the *Botryosphaeriaceae* are widespread fungal pathogens responsible for economically important diseases in woody plants. Despite the relevance of conidia production for understanding pathogen biology, infection processes, and disease epidemiology, sporulation *in vitro* remains unpredictable and inconsistent across species. In this study, we evaluated the efficacy of Vogel’s Minimal Medium (VMM) for inducing pycnidial and conidial development, which has recently been shown to be effective for this purpose in *Diplodia sapinea*, in species of *Diplodia* (n = 3), *Lasiodiplodia* (n = 2), and *Neofusicoccum* (n = 26). For this purpose, we used 123 isolates recently collected in the Canary Islands (Spain), as well as 67 *Neofusicoccum* isolates from long-term storage. All isolates were identified through multilocus phylogenetic analysis. The results showed that *D. africana*, *D. mutila*, and *D. seriata* were able to produce pycnidia, although only *D. seriata* consistently released conidia. *Lasiodiplodia brasiliensis* and *L. theobromae* successfully formed pycnidia and released conidia, while 20 of the 26 tested *Neofusicoccum* species formed pycnidia, of which 15 released conidia. Significant variation was observed in the time required for pycnidial development and conidial release, as well as in the quantity of conidia produced, both among and within species. Reduced sucrose concentration in VMM delayed pycnidia formation and conidial release and reduced sporulation yields in *Neofusicoccum* species but increased sporulation in *D. africana*, *D. seriata*, *L. brasiliensis*, and *L. theobromae*. Long-term storage on Malt Yeast Agar medium at 4 °C negatively affected sporulation in some species, including *N. luteum* and *N. stellenboschiana*. Overall, VMM provides a standardised and reproducible medium for inducing sporulation in the *Botryosphaeriaceae*, although notable variation persists within and between species. These findings provide a methodological foundation for future studies on the biology, pathogenicity, molecular biology, and host-pathogen interactions of these fungi.

## Introduction

Sporulation plays an essential role in the survival, dissemination, and infection strategies of fungi. These reproductive structures are often highly resistant to desiccation and other environmental stresses, allowing pathogens to survive in soil, on plant surfaces, or within plant tissues for extended periods (Carlile et al. 2001; Huang and Hull 2017). Sporulation also facilitates the ability of pathogens to infect new hosts and their geographic dispersal, which is critical for the epidemiology of plant diseases (West 2014). Studying spore morphology has therefore contributed significantly to understanding fungal biology and ecology.

Spore morphology reflects ecological adaptations that influence dispersal, host interactions, and infection efficiency. Hydrophobic spores adhere more effectively to plant surfaces, while wind-dispersed types dominate open fields, and mucilaginous spores are linked to humid environments (Braun and Howard 1994; Hughes et al. 1999; Calhim et al. 2018; Wang et al. 2021). Beyond dispersal, spores initiate infection by attaching to the plant cuticle through extracellular mucilage enriched with polysaccharides, glycoproteins, and enzymes such as cutinases (Hamer et al. 1988; Mendgen and Deising 1993; Arya et al. 2021). Their morphology can also aid in evading plant immunity, contributing to virulence (Almeida et al. 2019). Thus, spores function not only as propagules but also as active agents in host colonisation – a key consideration for disease control strategies.

Spore size, shape, and texture have historically played an important role in fungal taxonomy (Phillips et al. 2013). Although molecular resources are now essential for accurate species delimitation in fungi, morphological characters remain useful for preliminary identification, including in the *Botryosphaeriaceae*, which are the focus of this study (Slippers et al. 2017). Small but characteristic variations in the morphology of conidia provide useful diagnostic features at the genus and, in some cases, species level. For example, *Diplodia* species usually produce ellipsoidal conidia that become dark-walled and may develop fine longitudinal striations; *Lasiodiplodia* species often have large, thick-walled conidia with prominent longitudinal grooves; whereas *Neofusicoccum* species generally form fusiform, hyaline conidia that may become pigmented with age (Crous et al. 2006; Phillips et al. 2013; Zhang et al. 2021). Such characteristics may, however, overlap among species, and their usefulness for identification is therefore limited to scenarios in which the taxa present in a given environment are limited and already known.

The *Botryosphaeriaceae* (*Botryosphaeriales*, *Dothideomycetes*) comprise a diverse assemblage of endophytes and opportunistic pathogens associated with woody plants worldwide. Their taxonomy has historically been challenging because many species exhibit overlapping morphological characters and pronounced pleomorphism, with sexual morphs rarely encountered for many taxa and asexual morphs traditionally treated under separate generic names. Modern classifications have therefore relied heavily on multilocus phylogenetic frameworks, which have stabilised generic concepts and enabled robust species delimitation, while also revealing complexes of cryptic species within morphologically conserved groups. Consequently, reliable identification in the *Botryosphaeriaceae* is now typically based on analyses combining the internal transcribed spacer region (ITS1–5.8S–ITS2; ITS), the translation elongation factor 1-alpha gene (*tef*1-α), the beta-tubulin gene (*tub*2), and, for some genera, the DNA-directed RNA polymerase II second-largest subunit gene (*rpb*2), supported by curated reference datasets and ex-type or representative sequences, rather than morphology alone (Crous et al. 2006; Phillips et al. 2013; Slippers et al. 2013, 2017; Garcia et al. 2021). This taxonomic framework is particularly important when comparing phenotypes across closely related species and genera.

Members of the *Botryosphaeriaceae* typically sporulate on necrotic plant tissues, producing pycnidiospores (Slippers and Wingfield 2007; Úrbez-Torres 2011). Although the sexual morph has been described, it is rarely observed in nature (Phillips et al. 2012; Obrador-Sánchez and Hernández-Martínez 2020). Sporulation is influenced by multiple abiotic factors such as temperature, humidity, and nutrient availability, which are often species-specific and difficult to predict (Copes and Hendrix 2004; Vieira et al. 2021). Dispersal of these spores is mainly driven by wind, rain splash, and insect activity (Ahimera et al. 2004; Amponsah et al. 2009; Úrbez-Torres et al. 2010b; Billones-Baaijens et al. 2018).

In the *Botryosphaeriaceae*, studies specifically focused on spore traits or laboratory-based sporulation protocols remain limited. Nevertheless, several investigations have provided insights into the physiological and environmental factors regulating conidial production and germination. For instance, the effects of moisture and temperature have been explored for sporulation of *Botryosphaeria dothidea*, showing that conidial production is markedly enhanced under high humidity and is optimal at warm temperatures (25–30 °C), whereas drier conditions or suboptimal temperatures significantly reduce sporulation (Sutton and Arauz 1991). Úrbez-Torres et al. (2010a) assessed conidial germination across temperature gradients and found that optimal ranges varied among species. Further, Sammonds et al. (2019) demonstrated that conidia of *N. luteum*, *N. parvum*, and *B. dothidea* germinated equally on hydrophobic and hydrophilic surfaces, with germination enhanced by the presence of cellulose. Collectively, these findings underscore the complex interplay of abiotic cues in modulating sporulation and germination, while also highlighting the methodological challenges associated with standardising spore production. Despite their ecological and epidemiological importance, such investigations remain scarce in the *Botryosphaeriaceae*, partly due to difficulties in inducing sporulation under laboratory conditions.

*Botryosphaeriaceae* species do not regularly sporulate on conventional media such as Malt Extract Agar (MEA) or Potato Dextrose Agar (PDA). Accordingly, many culture media and incubation regimes have been tested in attempts to induce sporulation (Phillips 2016; Vieira et al. 2021). Among the most commonly used media is water agar supplemented with plant material, particularly pine needles, which stimulate pycnidial production by providing organic compounds and nutrients (Smith et al. 1996; Crous et al. 2006, 2019). In addition to pine needles, other substrates such as poplar twigs (González-Domínguez et al. 2017) and pistachio leaves (Chen et al. 2014; Agustí-Brisach et al. 2019) have also been used successfully. Although *in vitro* culture-based protocols are most common, alternative methods such as direct inoculation of plant tissues with mycelial plugs, e.g. green grapevine shoots, have also proven effective (Amponsah et al. 2011). The success and number of spores obtained using these methods are variable, possibly due to the inherently variable nature of host material derived from different species and ecological contexts.

A significant recent breakthrough in sporulation of the *Botryosphaeriaceae* was reported by Oostlander et al. (2023), who demonstrated that *D. sapinea* readily sporulates on Vogel’s Minimal Medium (VMM), producing large numbers of pycnidia within three weeks of incubation at 25 °C under constant white light. The medium was originally developed by Vogel (1956) for the growth of *Neurospora crassa* and is commonly used to induce its asexual sporulation (Greenwald et al. 2010). Oostlander et al. (2023) successfully induced the production of 1.7 × 10^6^ conidia mL^-1^ per Petri dish after 21 days of incubation. These conidia could be used for inoculation of seedlings, as well as for transformation. This technique not only regularly produces large numbers of spores but also provides a more standardised medium for comparing experiments. More recently, Slippers et al. (2024) demonstrated that the same method was also effective for *Oblongocollomyces ednahkunjekuae*.

In this study, we aimed to test the protocol developed by Oostlander et al. (2023) on three widespread genera within the *Botryosphaeriaceae*, namely *Diplodia*, *Lasiodiplodia*, and *Neofusicoccum*, which are important pathogens of woody plants worldwide (Crous et al. 2006; Batista et al. 2021; Garcia et al. 2021). We evaluated the ability of VMM to induce sporulation in three species of *Diplodia*, two species of *Lasiodiplodia*, and 26 species of *Neofusicoccum*. Additionally, we evaluated different sucrose concentrations in the VMM formulation to determine optimal conditions for sporulation among species and isolates, and we assessed whether long-term storage on Malt Yeast Agar medium influenced sporulation of *Neofusicoccum* spp. on this medium.

## Methods

### Fungal isolates and identification

The efficacy of VMM in supporting sporulation of *Botryosphaeriaceae* species was evaluated using 123 isolates from the fungal collection of the Instituto Canario de Investigaciones Agrarias (Canary Islands, Spain). All strains were obtained between 2021 and 2024 from a range of hosts, including *Delonix regia*, *Dracaena draco*, *Ficus macrocarpa*, *Persea americana* Mill., *Phoenix canariensis*, *Prunus domestica*, *Pinus radiata*, *Rosa* sp., *Schinus molle*, *Schotia brachypetala*, and *Vitis vinifera*, and some have been previously reported by Hernández et al. (2023) (Suppl. material 1: table SS1). Taxonomic authorities for the taxa identified in this study are provided at first mention in the Results. Species-level identification of all isolates was based on sequencing four loci: the internal transcribed spacer regions 1 and 2, including the intervening 5.8S nrDNA gene (ITS), the translation elongation factor 1-alpha gene (*tef*1-α), the beta-tubulin gene (*tub*2), and the DNA-directed RNA polymerase II second-largest subunit gene (*rpb*2). Primers used were ITS1/ITS4 (White et al. 1990), EF1-728F/EF1-986R (Carbone and Kohn 1999), Bt2a/Bt2b (Glass and Donaldson 1995), RPB2-LasF/RPB2-LasR (Cruywagen et al. 2017) for *Lasiodiplodia* species, and RPB2bot6F/RPB2bot7R (Sakalidis et al. 2011) for *Neofusicoccum* species. PCR amplification protocols followed those described by Pavlic et al. (2007), Phillips et al. (2013), Slippers et al. (2013), and Cruywagen et al. (2017). Maximum likelihood and Bayesian phylogenetic analyses were performed using RAxML-HPC2 v.8.2.12 (Stamatakis 2014) and MrBayes v.3.2.7 (Ronquist et al. 2012), respectively, and were executed via the CIPRES Science Gateway (Miller et al. 2010) using representative and type sequences from all species of the *Diplodia*, *Lasiodiplodia*, and *Neofusicoccum* genera, as described in Hernández et al. (2023).

### Evaluation of VMM for stimulating pycnidial and conidial formation

All strains were initially grown on 2% Malt Extract Agar (MEA; Condalab #1708.25) plates for one week at 22 ± 1 °C. Subsequently, mycelial plugs were transferred to VMM plates (two plates per isolate) prepared according to Oostlander et al. (2023), with the agar concentration increased to 2%. Plates were sealed with Parafilm® and incubated under constant white light (Sylvania, F36W/T8/ACTIVA, 0002219) at 25 °C. The distance between the light source and the surface of the VMM plates was 52 cm. All plates were positioned with the mycelial surface facing upwards to ensure direct light exposure. Initial pycnidia formation and first conidial discharge were recorded daily. Plates were maintained in incubation until peak sporulation was reached for each isolate, for a maximum of 35 days. Upon observing conidial release, primarily in cirrus form, strains producing abundant and homogeneous cirri across the entire plate, in most species, were used to determine the conidial concentration produced under the specified incubation conditions. The incubation time required for spore release to allow conidial harvesting was also recorded. The protocol developed by Oostlander et al. (2023) for harvesting conidia was followed with the following modifications: instead of pipetting 2 mL of Tween 20 solution (0.01% v/v in distilled water) onto the surface of each culture, the solution was added until the entire surface of the plate was covered, ensuring that all pycnidia and cirri were submerged. Each plate was incubated at 22 ± 1 °C for at least 30 minutes, with gentle shaking every 10 minutes to dislodge conidia from the pycnidia. Plates were then rinsed several times with the Tween solution, and the suspension was transferred to a 15 mL tube. Tubes were centrifuged for 15 minutes at 4700 rpm to obtain a conidial pellet. The Tween solution was removed, and the pellet was resuspended in 30% glycerol and transferred to a new 1.5 mL tube. The concentration of each conidial suspension was measured twice using a Neubauer-improved counting chamber. The resulting suspensions were stored at −80 °C, and spore viability was confirmed one month later by plating an aliquot on MEA to verify spore germination and subsequent mycelial growth.

### Effect of sucrose concentration on pycnidial development and conidial release

In certain species, such as *N. parvum*, pycnidia may develop beneath dense aerial mycelium or become embedded within the culture medium, which can hinder efficient conidial harvesting. In other cases, pycnidia formation is restricted to the oldest area of the culture or to the edges of the colony on the Petri dish. To determine whether these features could be modified, the concentration of sucrose in VMM was adjusted. The effect of sucrose concentration on sporulation was evaluated over a 7-week period for *Neofusicoccum* species, 3 weeks for *Lasiodiplodia* species, and 2–5 weeks for *Diplodia* species, reflecting the time required for isolates of each genus to reach peak sporulation. Variation in assay duration among species was based on weekly macroscopic evaluations of each culture, which were used to determine the point at which each isolate reached peak sporulation. The recommended sucrose concentration in VMM (1.5% w/v) was adjusted to 1%, 0.5%, 0.25%, and 0%. A total of 18 representative isolates were selected for this assay: *D. africana* (n = 2), *D. mutila* (n = 1), *D. seriata* (n = 2), *Lasiodiplodia brasiliensis* (n = 2), *L. theobromae* (n = 1), *N. australe* (n = 1), *N. cryptoaustrale* (n = 1), *N. luteum* (n = 2), *N. parvum* (n = 4), and *N. stellenboschiana* (n = 2) (Suppl. material 1: table SS1). All cultures were incubated under the conditions described above. The onset of pycnidial formation and first conidial discharge were recorded daily, together with the incubation time required to achieve sufficient sporulation for conidial harvesting and concentration measurements.

### Sporulation of *Neofusicoccum* strains in long-term storage

A total of 67 strains representing 26 *Neofusicoccum* species were selected from the Culture Collection of the Forestry and Agricultural Biotechnology Institute (FABI) (CMW) at the University of Pretoria, South Africa. The cultures were stored on Malt Yeast Agar (MYA; 3 g malt extract, 3 g yeast extract, 10 g glucose, 5 g peptone, and 25 g agar) slants at 4 °C. This assay aimed to determine whether the findings on pycnidia formation and conidial discharge in recently isolated *Neofusicoccum* strains from the Canary Islands, as described above, were applicable to a broader range of strains, species, hosts, and geographic origins, and to assess the potential impact of long-term storage on their ability to sporulate on VMM. Strains were initially transferred from the collection to MEA plates and incubated at 22 ± 1 °C for one week. These MEA plates were retained for 2 weeks after transfer to VMM to confirm pycnidia formation or conidial exudation. Subsequently, each strain was subcultured onto two VMM plates and incubated under the same conditions described above. Initial pycnidia formation and first conidial discharge were recorded daily, along with the incubation time required for conidial harvest and the concentration of conidia obtained, following the same protocol described previously. In addition, genomic DNA was extracted from all isolates, and the ITS region was amplified using primers ITS1 and ITS4, as described above. The obtained amplicons were sequenced at the University of Pretoria Sequencing Facility to confirm that all isolates were uncontaminated and belonged to the genus *Neofusicoccum*.

## Results

### Fungal isolates and identification

**Figure 1.**
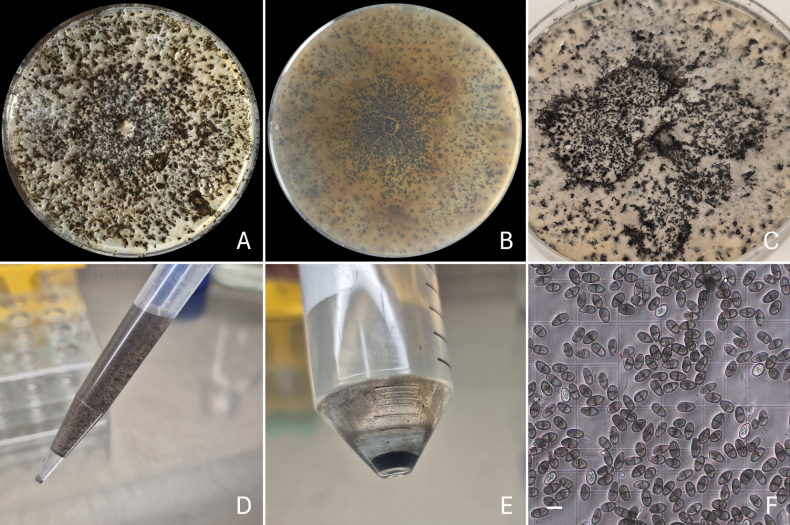
Harvesting conidia from *Lasiodiplodia brasiliensis* (D106 = CMW 67234) incubated on VMM for 21 days. **A, B** Top and bottom views of the culture; **C** Culture immersed in a solution of 0.01% (v/v) Tween 20; **D** Conidial suspension recovered from the culture; **E** Conidia pellet after centrifugation; **F** Quantification of conidia under a light microscope. Scale bar: 20 μm.

**Figure 2.**
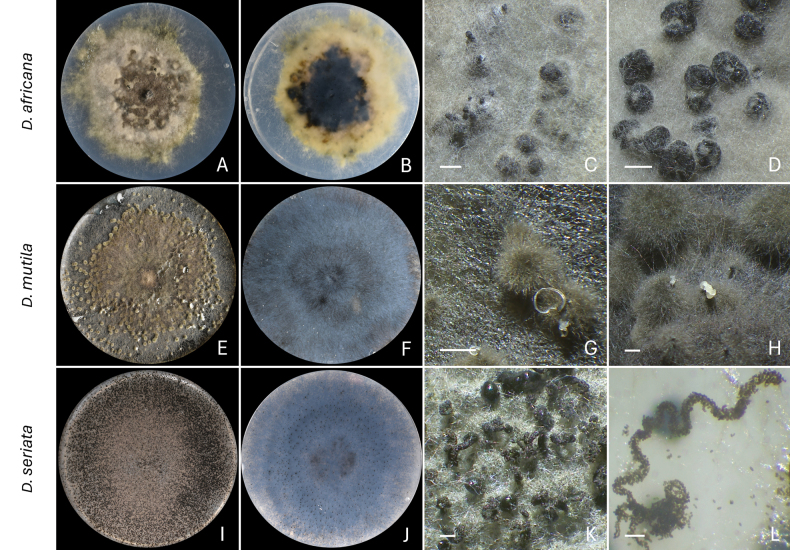
Sporulation of *Diplodia* species. **A–D***D. africana* (D242 = CMW 67242) after 21 days of incubation; **E–H***D. mutila* (D120 = CMW 67239) after 21 days of incubation; **I–L***D. seriata* (13.1.1 = CMW 67254) after 14 days of incubation. **A, E, I** Top view of the culture; **B, F, J** Bottom view of the culture; **C, D** Pycnidia; **G, H, K** Pycnidia releasing conidia in the form of cirri; **L** Cirrus released from a pycnidium. Scale bars: 0.4 mm (**C–D**); 0.5 mm (**G–H**); 1 mm (**K**); 0.1 mm (**L**).

**Figure 3.**
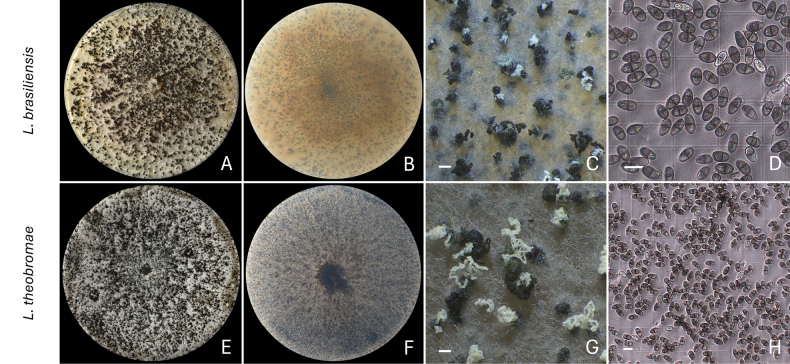
Sporulation of *Lasiodiplodia* species after 14 days of incubation. **A–D***L. brasiliensis* (D106 = CMW 67234); **E–H***L. theobromae* (D113 = CMW 67235). **A, E** Top view of the culture; **B, F** Bottom view of the culture; **C–G** Pycnidia releasing conidia in the form of cirri; **D, H** Quantification of conidia under a light microscope. Scale bars: 1 mm (**C, G**); 20 μm (**D, H**).

The phylogenetic analyses based on the ITS, *tef*1-α, *tub*2, and *rpb*2 loci enabled the identification of all 123 isolates at the species level (Suppl. material 1: tables S1, S3–S5, figs 1–3). Isolates were assigned to ten *Botryosphaeriaceae* species: *Diplodia africana* Damm & Crous (n = 12), *D. mutila* (Fr.) Fr. (n = 1), *D. seriata* De Not. (n = 2), *Lasiodiplodia brasiliensis* M.S.B. Netto, M.W. Marques & A.J.L. Phillips (n = 11), *L. theobromae* Griffon & Maublanc (n = 1), *Neofusicoccum australe* (Slippers, Crous & M.J. Wingfield) Crous, Slippers & A.J.L. Phillips (n = 1), *N. cryptoaustrale* Pavlic, Maleme, Slippers & M.J. Wingfield (n = 1), *N. luteum* (Pennycook & Samuels) Crous, Slippers & A.J.L. Phillips (n = 23), *N. parvum* (Pennycook & Samuels) Crous, Slippers & A.J.L. Phillips (n = 37), and *N. stellenboschiana* Tao Yang & Crous (n = 34). Forty-seven of these isolates had been preliminarily identified and reported by Hernández et al. (2023), while the remaining 76 isolates were identified for the first time in this study.

### Evaluation of VMM for stimulating pycnidial and conidial formation

**Figure 4.**
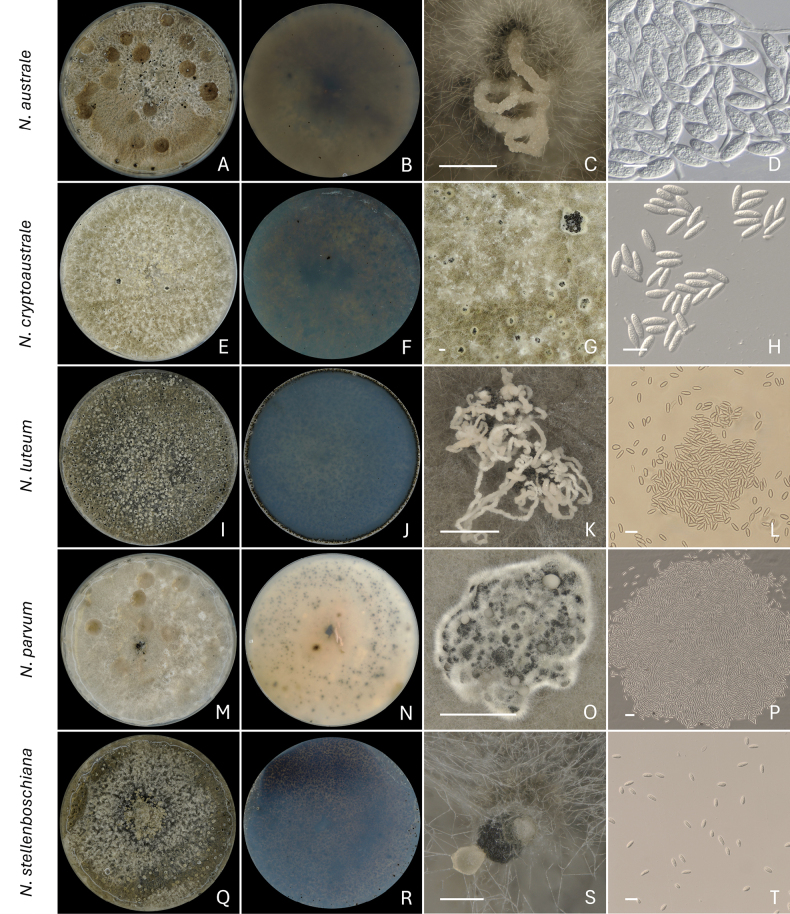
Sporulation of *Neofusicoccum* species. **A–D***N. australe* (D31 = B018 = CMW 64262) after 28 days of incubation; **E–H***N. cryptoaustrale* (D39 = B043 = CMW 64270) after 35 days of incubation; **I–L***N. luteum* (D89 = CMW 64314) after 21 days of incubation; **M–P***N. parvum* (D50 = B110 = CMW 64281) after 28 days of incubation; **Q–T***N. stellenboschiana* (D43 = B050 = CMW 64274) after 28 days of incubation. **A, E, I, M, Q** Top view of the culture; **B, F, J, N, R** Bottom view of the culture; **C, K, O, S** Pycnidia releasing conidia in the form of cirrus; **D, H, L, P, T** Conidia; **G** Pycnidia. Scale bars: 1 mm (**C, G**); 20 μm (**D, H, L, P, T**); 0.5 mm (**K, O, S**).

To assess the capacity of VMM to promote sporulation, 123 isolates were initially evaluated (Fig. 1). Of these, 50 isolates that produced abundant and homogeneous cirri across the entire plate were selected to determine the conidial concentration that each species could produce under the specified incubation conditions (Suppl. material 1: table SS2). The results obtained with the selected isolates of *Diplodia* (Fig. 2), *Lasiodiplodia* (Fig. 3), and *Neofusicoccum* (Fig. 4) confirmed the effectiveness of VMM in inducing pycnidia formation across a broad range of species within these genera (Table 1). All isolates of *D. mutila*, *D. seriata*, *L. theobromae*, *N. australe*, *N. cryptoaustrale*, and *N. luteum* produced pycnidia. Additionally, pycnidia were observed in 91.7% of *D. africana*, 81.8% of *L. brasiliensis*, 83.8% of *N. parvum*, and 97.1% of *N. stellenboschiana* isolates. The mean time to pycnidia formation varied among species, occurring at 3 days for *D. seriata*; 4 days for *N. cryptoaustrale* and *N. luteum*; 5 days for *L. brasiliensis*, *L. theobromae*, *N. australe*, *N. parvum*, and *N. stellenboschiana*; and 14 days for *D. mutila* and *D. africana*.

**Table 1.** Sporulation dynamics data of *Botryosphaeriaceae* species subcultured onto VMM.

Species	Isolates	Isolates with pycnidia formation	Isolates with conidial discharge	Mean days to initial pycnidia formation	Mean days to initial conidial discharge	Mean days to conidia harvest	Mean conidial concentration (conidia mL^-1^)
***Diplodia africana***	12	11 (91.7%)	5 (41.7%)	14	18	35	0
***D. mutila***	1	1 (100%)	1 (100%)	14	21	35	0
***D. seriata***	2	2 (100%)	2 (100%)	3	7	14	1.3 × 10^6^
***Lasiodiplodia brasiliensis***	11	9 (81.8%)	9 (81.8%)	5	6	21	8.7 × 10^6^
***L. theobromae***	1	1 (100%)	1 (100%)	5	6	21	7.12 × 10^6^
***Neofusicoccum australe***	1	1 (100%)	1 (100%)	5	14	28	3.75 × 10^5^
***N. cryptoaustrale***	1	1 (100%)	1 (100%)	4	25	35	1.25 × 10^5^
***N. luteum***	23	23 (100%)	23 (100%)	4	7	21	1.5 × 10^7^
***N. parvum***	37	31 (83.8%)	22 (59.5%)	5	21	35	2.31 × 10^6^
***N. stellenboschiana***	34	33 (97.1%)	26 (76.5%)	5	21	35	2 × 10^6^

0: no conidia were observed under the microscope.

All isolates of *D. mutila*, *D. seriata*, *L. theobromae*, *N. australe*, *N. cryptoaustrale*, and *N. luteum* released conidia in the form of cirri. However, not all isolates of *D. africana*, *L. brasiliensis*, *N. parvum*, and *N. stellenboschiana* were able to release conidia from the pycnidia, and those isolates that sporulated did so by releasing their conidia in the form of cirri (Table 1). Only 41.7% of *D. africana* isolates released conidia, while 81.8%, 59.5%, and 76.5% of *L. brasiliensis*, *N. parvum*, and *N. stellenboschiana* isolates were able to do so, respectively. Species such as *D. seriata*, *L. brasiliensis*, *L. theobromae*, and *N. luteum* produced the most abundant and homogeneous cirri across the entire culture. The time required to observe the first conidial discharge differed among species. The first species to release conidia after being subcultured onto VMM were *L. brasiliensis* and *L. theobromae*, followed by *D. seriata* and *N. luteum*. Subsequently, *N. australe* started to release conidia, whereas *D. africana*, *D. mutila*, *N. parvum*, *N. stellenboschiana*, and *N. cryptoaustrale* were the last to do so.

The decision on the optimal time for harvesting conidia, from the day of subculturing onto VMM, was based on the cessation of new pycnidia and cirri formation. Each culture was monitored daily until no further pycnidia or cirri were produced. Consequently, the optimal time for conidia harvesting ranged from 14 days for the earliest species to 35 days for the slowest (Table 1). *D. africana*, *D. mutila*, *N. cryptoaustrale*, *N. parvum*, and *N. stellenboschiana* required the longest time for conidia collection and also showed the greatest delay in initiating conidial release from pycnidia. Species such as *N. parvum* and *N. stellenboschiana* exhibited prominent mycelial development, particularly *N. parvum*, which concealed and delayed the sporulation process, resulting in a delay in conidia harvesting. In the case of *D. africana*, pycnidia were produced exclusively in the oldest area of the culture, around the 5-mm mycelial plug placed onto the VMM plate.

With regard to the mean number of conidia yielded (Table 1), *N. luteum* generated the highest concentration, followed by *L. brasiliensis* and *L. theobromae*. Although *N. parvum* and *N. stellenboschiana* required the longest time for conidia harvest, these species yielded concentrations of up to 2 × 10^6^ conidia mL^-1^. The only *Diplodia* species for which conidial concentration could be quantified was *D. seriata*. The lowest conidia production was recorded for *N. australe* and *N. cryptoaustrale*, with less than 4 × 10^5^ conidia mL^-1^ for both species. Although pycnidia and conidia were macroscopically observed in *D. africana* and *D. mutila*, no conidia were detected using the Neubauer-improved counting chamber.

### Effect of sucrose concentration on pycnidia development and conidia release

**Figure 5.**
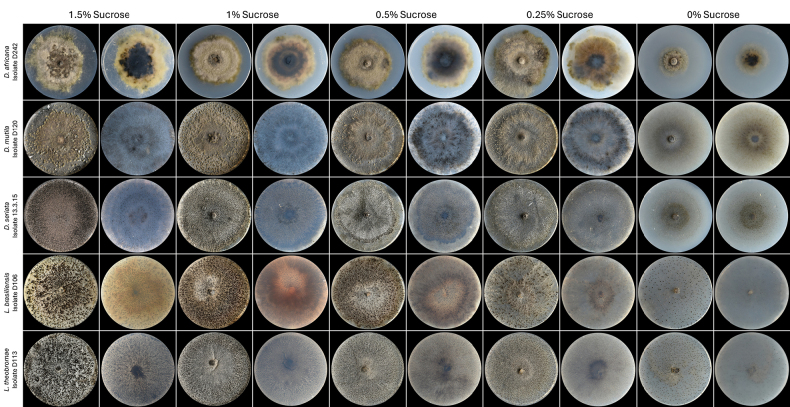
Colony morphology of *Diplodia* and *Lasiodiplodia* species used in the sucrose-concentration assay on pycnidia development and conidial release. Each photograph was taken prior to conidial harvesting. See Table 2 for harvest dates.

**Figure 6.**
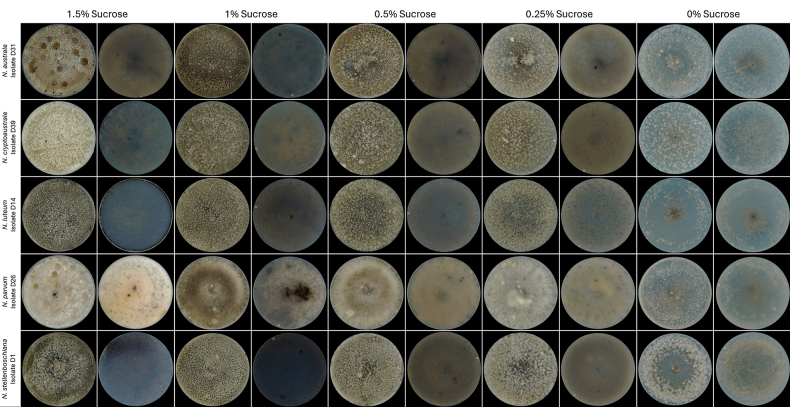
Colony morphology of *Neofusicoccum* species used in the sucrose-concentration assay on pycnidia development and conidial release. Each photograph was taken prior to conidial harvesting. See Table 2 for harvest dates.

Daily macroscopic evaluation of all species (Figs 5, 6) revealed that, although all tested species developed pycnidia under most conditions, their ability to release conidia varied significantly, depending on the species and sucrose availability. In general, higher or intermediate sucrose concentrations (particularly 1.5% and 1%) promoted earlier development and greater sporulation in most species, whereas low or absent sucrose levels often delayed pycnidia formation and inhibited or entirely suppressed conidial release, especially in species such as *D. africana*, *D. mutila*, and *N. cryptoaustrale* (Table 2).

**Table 2.** Effect of sucrose concentration on sporulation in VMM.

Species (n° isolates)	Sucrose concentration in VMM (%)				
***Diplodia africana*** (2)	**1.5%**	**1%**	**0.5%**	**0.25%**	**0%**
Initial pycnidia formation	14 d	8 d	9 d	21 d	21 d
First conidial discharge	18 d	14 d	NC	NC	28 d
Conidial harvest	35 d	35 d	35 d	35 d	35 d
Mean conidial concentration across all selected isolates (conidia mL^-1^)	0	0	0	0	7.5 × 10^4^
***D. mutila*** (1)	**1.5%**	**1%**	**0.5%**	**0.25%**	**0%**
Initial pycnidia formation	14 d	7 d	8 d	14 d	21 d
First conidial discharge	21 d	14 d	14 d	NC	NC
Conidial harvest	35 d	28 d	28 d	28 d	28 d
Mean conidial concentration across all selected isolates (conidia mL^-1^)	0	0	0	0	0
***D. seriata*** (2)	**1.5%**	**1%**	**0.5%**	**0.25%**	**0%**
Initial pycnidia formation	3 d	5 d	5 d	5 d	7 d
First conidial discharge	7 d	7 d	7 d	7 d	10 d
Conidial harvest	14 d	14 d	14 d	14 d	14 d
Mean conidial concentration across all selected isolates (conidia mL^-1^)	1.3 × 10^6^	9.29 × 10^6^	1.36 × 10^7^	1.07 × 10^7^	3.37 × 10^5^
***Lasiodiplodia brasiliensis*** (2)	**1.5%**	**1%**	**0.5%**	**0.25%**	**0%**
Initial pycnidia formation	5 d	3 d	3 d	3 d	4 d
First conidial discharge	6 d	7 d	7 d	7 d	7 d
Conidial harvest	21 d	21 d	21 d	21 d	21 d
Mean conidial concentration across all selected isolates (conidia mL^-1^)	8.7 × 10^6^	1.44 × 10^7^	1.09 × 10^7^	1.28 × 10^7^	1.84 × 10^6^
***L. theobromae*** (1)	**1.5%**	**1%**	**0.5%**	**0.25%**	**0%**
Initial pycnidia formation	5 d	3 d	3 d	3 d	3 d
First conidial discharge	6 d	7 d	7 d	7 d	7 d
Conidial harvest	21 d	21 d	21 d	21 d	21 d
Mean conidial concentration across all selected isolates (conidia mL^-1^)	7.12 × 10^6^	2.01 × 10^7^	9.25 × 10^6^	9.18 × 10^6^	1.83 × 10^6^
***Neofusicoccum australe*** (1)	**1.5%**	**1%**	**0.5%**	**0.25%**	**0%**
Initial pycnidia formation	5 d	6 d	10 d	10 d	14 d
First conidial discharge	14 d	14 d	14 d	14 d	21 d
Conidial harvest	28 d	21 d	28 d	49 d	49 d
Mean conidial concentration across all selected isolates (conidia mL^-1^)	3.75 × 10^5^	2.7 × 10^6^	6.25 × 10^5^	0	0
***N. cryptoaustrale*** (1)	**1.5%**	**1%**	**0.5%**	**0.25%**	**0%**
Initial pycnidia formation	4 d	6 d	6 d	6 d	10 d
First conidial discharge	21 d	21 d	21 d	28 d	NC
Conidial harvest	21 d	49 d	49 d	49 d	49 d
Mean conidial concentration across all selected isolates (conidia mL^-1^)	1.25 × 10^5^	3 × 10^5^	1.75 × 10^5^	0	0
***N. luteum*** (2)	**1.5%**	**1%**	**0.5%**	**0.25%**	**0%**
Initial pycnidia formation	4 d	4 d	6 d	6 d	6 d
First conidial discharge	7 d	7 d	7 d	7 d	14 d
Conidial harvest	21 d	21 d	35 d	35 d	35 d
Mean conidial concentration across all selected isolates (conidia mL^-1^)	1.5 × 10^7^	7.21 × 10^6^	2.05 × 10^6^	1.7 × 10^6^	0
***N. parvum*** (4)	**1.5%**	**1%**	**0.5%**	**0.25%**	**0%**
Initial pycnidia formation	5 d	6 d	6 d	6 d	17 d
First conidial discharge	21 d	21 d	21 d	21 d	35 d
Conidial harvest	28-42 d	35-49 d	35-49 d	35-49 d	35-49 d
Mean conidial concentration across all selected isolates (conidia mL^-1^)	2.31 × 10^6^	9 × 10^5^	6.56 × 10^5^	3.17 × 10^5^	0
***N. stellenboschiana*** (2)	**1.5%**	**1%**	**0.5%**	**0.25%**	**0%**
Initial pycnidia formation	5 d	6 d	6 d	6 d	10 d
First conidial discharge	21 d	21 d	21 d	21 d	21 d
Conidial harvest	28-42 d	49 d	49 d	49 d	49 d
Mean conidial concentration across all selected isolates (conidia mL^-1^)	2 × 10^6^	1.03 × 10^6^	1.25 × 10^6^	3.5 × 10^5^	0

**d**, days; **NC**, no conidia discharge.

*Diplodia africana* formed pycnidia at all sucrose levels, with conidia release occurring only at 1.5%, 1%, and 0%, although conidia were only quantified at 0% sucrose. In *D. mutila*, despite visible pycnidia development across treatments, no conidia were quantified, indicating high sensitivity to environmental or nutritional constraints. In contrast, *D. seriata* consistently released conidia under all sucrose-supplemented conditions, with optimal yields at 0.5% and 0.25%. Similarly, *L. brasiliensis* and *L. theobromae* responded favourably to intermediate concentrations, particularly 1%, which supported both early development and maximal conidia production, whereas 0% sucrose resulted in the lowest sporulation. Among *Neofusicoccum* spp., a clear preference for 1–1.5% sucrose was evident. For example, *N. australe* reached peak conidial production at 1%, whereas *N. luteum*, *N. stellenboschiana*, and *N. parvum* exhibited earlier and more prolific sporulation at 1.5%. Notably, *N. cryptoaustrale* displayed low productivity under all conditions, suggesting intrinsic physiological limitations under the experimental conditions (Table 3). Across all species, sucrose deprivation (0%) consistently resulted in delayed pycnidia development and minimal or absent conidial release.

**Table 3.** Optimal sucrose concentration for sporulation on VMM.

**Species**	**Optimal sucrose concentration (%)**
*D. africana*	0
*D. mutila*	–
*D. seriata*	0.5–0.25
*L. brasiliensis*	1
*L. theobromae*	1
*N. australe*	1
*N. cryptoaustrale*	1
*N. luteum*	1.5
*N. parvum*	1.5
*N. stellenboschiana*	1.5

### Sporulation of *Neofusicoccum* strains in long-term storage

From the 26 selected *Neofusicoccum* species (taxonomic authorities are provided in Table 4) preserved in the CMW collection (Table 4), 20 develo­ped pycnidia on VMM (Table 5). The species for which all isolates failed to produce pycnidia under these conditions were *N. andinum*, *N. kwambonambiense*, *N. luteum*, *N. mangiferae*, *N. stellenboschiana*, and *N. terminaliae*. The time required for first visible pycnidial formation among the remaining species ranged from 3 to 38 days. Some isolates from 12 species (*N. australe*, *N. cryptoaustrale*, *N. dianense*, *N. eucalypticola*, *N. eucalyptorum*, *N. lumnitzerae*, *N. microconidium*, *N. ningerense*, *N. occulatum*, *N. pandanicola*, *N. variabile*, and *N. vitifusiforme*) discharged conidia, producing visible cirri from pycnidia. The incubation time required for the first conidial discharge ranged from 18 to 32 days.

**Table 4.** Origin and GenBank accession numbers of 67 *Neofusicoccum* strains from the CMW collection.

**Species name**	**Isolate**	**Host**	**Country**	**Collector**	**Isolation year**	**Reference**	**GenBank accession numbers**			
							**ITS**	***tef1***	***tub2***	***rpb2***
***Neofusicoccum andinum*** Mohali, Slippers & M.J. Wingfield	CMW 13446	*Eucalyptus* sp.	Venezuela	S. Mohali	2003	Mohali et al. (2006)	DQ306263	DQ306264	KX464922	KX464001
	**CMW 13455**	*Eucalyptus* sp.	Venezuela	S. Mohali	2003	Mohali et al. (2006)	AY693976	AY693977	KX464923	KX464002
***Neofusicoccum australe*** (Slippers, Crous & M.J. Wingfield) Crous, Slippers & A.J.L. Phillips	CMW 821	*Acacia mearnsii*	South Africa	M.J. Morris	1986	Vu et al. (2019)	MH863758	MT592170	MT592662	MT592359
	CMW 1133	*Plum*	South Africa	A. Smith	1996	Slippers et al. (2007)	DQ836716	MT592168	MT592660	MT592357
	CMW 1187	*Almond* sp.	South Africa	A. Smith	1998	Slippers et al. (2007)	DQ836718	MT592169	MT592661	MT592358
	**CMW 6837**	*Acacia* sp.	Australia	M.J. Wingfield	2000	Slippers et al. (2004b)	AY339262	AY339270	AY339254	EU339573
	CMW 6838	*Acacia* sp.	Australia	M.J. Wingfield	2000	Zhang et al. (2021)	MT587455	MT592164	MT592656	MT592352
	CMW 6853	*Sequoiadendron giganteum*	Australia	M.J. Wingfield	2000	Slippers et al. (2004b)	AY339263	AY339271	AY339255	MT592353
	CMW 9073	*Acacia* sp.	Australia	J. Roux & D. Guest	1999	Slippers et al. (2004b)	AY339261	AY339269	AY339253	MT592351
***Neofusicoccum batangarum*** Begoude, Jolanda Roux & Slippers	CMW 28320	*Terminalia catappa*	Cameroon	D. Begoude & J. Roux	2007	Begoude et al. (2010)	FJ900608	FJ900654	FJ900635	FJ900616
	**CMW 28363**	*Terminalia catappa*	Cameroon	D. Begoude & J. Roux	2007	Begoude et al. (2010)	FJ900607	FJ900653	FJ900634	FJ900615
***Neofusicoccum cordaticola*** Pavlic, Slippers & M.J. Wingfield	**CMW 13992**	*Syzygium cordatum*	South Africa	D. Pavlic	2002	Pavlic et al. (2009)	EU821898	EU821868	EU821838	EU821928
	CMW 14056	*Syzygium cordatum*	South Africa	D. Pavlic	2002	Pavlic et al. (2009)	EU821903	EU821873	EU821843	EU821933
***Neofusicoccum cryptoaustrale*** Pavlic, Maleme, Slippers & M.J. Wingfield	**CMW 23785**	*Eucalyptus tree*	South Africa	H.M. Maleme	2006	Crous et al. (2013)	FJ752742	FJ752713	FJ752756	KX464014
***Neofusicoccum dianense*** G.Q. Li & S.F. Chen	CMW 54123	*Eucalyptus* sp.	Indonesia	F. Jami	2018	Jami et al. (2022)	MT936368	MT949097	MZ564205	–
	CMW 54139	*Eucalyptus* sp.	Indonesia	F. Jami	2018	Jami et al. (2022)	MT936382	MT949112	MZ564220	–
	CMW 54189	*Eucalyptus* sp.	Indonesia	F. Jami	2018	Jami et al. (2022)	MT936405	MT949131	MZ564240	–
***Neofusicoccum eucalypticola*** (Slippers, Crous & M.J. Wingfield) Crous, Slippers & A.J.L. Phillips	CMW 6217	*Eucalyptus rossii*	Australia	M.J. Wingfield	2000	Slippers et al. (2004c)	AY615143	AY615135	AY615127	–
	CMW 6229	*Eucalyptus grandis*	Australia	M.J. Wingfield	2000	Slippers et al. (2004c)	AY615142	AY615134	AY615126	–
	**CMW 6539**	*Eucalyptus grandis*	Australia	M.J. Wingfield	2000	Slippers et al. (2004c)	AY615141	AY615133	AY615125	–
	CMW 6543	*Eucalyptus* sp.	Australia	M.J. Wingfield	2000	Slippers et al. (2004c)	AY615140	AY615132	AY615124	MT592378
***Neofusicoccum eucalyptorum*** (Crous, H. Sm. ter & M.J. Wingfield) Crous, Slippers & A.J.L. Phillips	CMW 6233	*Eucalyptus nitens*	Australia	M.J. Wingfield	2000	Slippers et al. (2004c)	AY615138	AY615130	AY615122	MT592381
	**CMW 10125**	*Eucalyptus grandis*	South Africa	H. Smith	2001	Smith et al. (2001)	AF283687	AY236892	AY236921	–
***Neofusicoccum kwambonambiense*** Pavlic, Slippers & M.J. Wingfield	**CMW 14023**	*Syzygium cordatum*	South Africa	D. Pavlic	2002	Pavlic et al. (2009)	EU821900	EU821870	EU821840	EU821930
	CMW 14140	*Syzygium cordatum*	South Africa	D. Pavlic	2003	Pavlic et al. (2009)	EU821919	EU821889	EU821859	EU821949
***Neofusicoccum lumnitzerae*** J.A. Osorio, Jolanda Roux & Z.W. de Beer	CMW 41228	*Lumnitzera racemosa*	South Africa	J. A. Osorio	2012	Osorio et al. (2016)	KP860882	KP860725	KP860803	KU587926
	CMW 41613	*Lumnitzera racemosa*	South Africa	J. A. Osorio	2011	Osorio et al. (2016)	KU587958	KU587948	KU587869	KU587924
	**CMW 41469**	*Lumnitzera racemosa*	South Africa	J. A. Osorio & J. Roux	2012	Osorio et al. (2016)	KP860881	KP860724	KP860801	KU587925
***Neofusicoccum luteum*** (Pennycook & Samuels) Crous, Slippers & A.J.L. Phillips	CMW 14071	*Syzygium cordatum*	South Africa	D. Pavlic	2002	Pavlic et al. (2007)	DQ316088	MT592196	MT592688	MT592387
***Neofusicoccum mangiferae*** (Syd. & P. Syd.) Crous, Slippers & A.J.L. Phillips	CMW 7797	*Mangifera indica*	Australia	G.I. Johnson	1988	Slippers et al. (2005)	AY615186	DQ093220	AY615173	KX464023
	**CMW 7024**	*Mangifera indica*	Australia	G.I. Johnson	1988	Slippers et al. (2005)	AY615185	DQ093221	AY615172	–
***Neofusicoccum mangroviorum*** J.A. Osorio, Jolanda Roux & Z.W. de Beer	**CMW 41365**	*Avicennia marina*	South Africa	J. A. Osorio & J. Roux	2012	Osorio et al. (2016)	KP860859	KP860702	KP860779	KU587905
	CMW 42355	*Lumnitzera racemosa*	South Africa	J. A. Osorio	2011	Osorio et al. (2016)	KP860879	KP860722	KP860799	MT592397
	CMW 42481	*Bruguiera gymnorrhiza*	South Africa	J. A. Osorio	2011	Osorio et al. (2016)	KP860848	KP860692	KP860770	KU587895
***Neofusicoccum microconidium*** G.Q. Li & S.F. Chen	CMW 13998	*Syzygium cordatum*	South Africa	D. Pavlic	2002	Pavlic et al. (2007)	DQ316081	MT592212	MT592704	MT592402
***Neofusicoccum ningerense*** G.Q. Li & S.F. Chen	CMW 54121	*Eucalyptus* sp.	Indonesia	F. Jami	2018	Jami et al. (2022)	MT936367	MT949096	MZ564204	–
	CMW 54144	*Eucalyptus* sp.	Indonesia	F. Jami	2018	Jami et al. (2022)	MT936387	MT949117	MZ564225	–
	CMW 54212	*Eucalyptus* sp.	Indonesia	F. Jami	Unknown	Jami et al. (2022)	MT936418	MT949144	MZ564253	–
***Neofusicoccum occulatum*** Sakalidis & T. Burgess	CMW 54187	*Eucalyptus* sp.	Indonesia	F. Jami	2018	Jami et al. (2022)	MT936403	MT949129	MZ564238	–
***Neofusicoccum pandanicola*** Tibpromma & K.D. Hyde	CMW 14029	*Syzygium cordatum*	South Africa	M.J. Wingfield	2002	Pavlic et al. (2007)	DQ316078	MT592216	MT592708	MT592405
	CMW 14087	*Syzygium cordatum*	South Africa	D. Pavlic	2002	Pavlic et al. (2009)	EU821909	EU821879	EU821849	EU821939
***Neofusicoccum parvum*** (Pennycook & Samuels) Crous, Slippers & A.J.L. Phillips	CMW 9071	*Ribes* sp.	Australia	M.J. Wingfield	2000	Slippers et al. (2004c)	AY236938	AY236880	AY236909	EU339571
	CMW 9080	*Populus nigra*	New Zealand	G.J. Samuels	2005	Slippers et al. (2004a)	AY236942	AY236887	AY236916	EU821962
	**CMW 9081**	*Populus nigra*	New Zealand	G.J. Samuels	2005	Slippers et al. (2004a)	AY236943	AY236888	AY236917	EU821963
	CMW 13350	*Guava* sp.	Venezuela	L. Cedeno	2002	Mohali et al. (2007)	EF118036	MT592241	MT592733	MT592430
***Neofusicoccum parvum*** (Pennycook & Samuels) Crous, Slippers & A.J.L. Phillips	CMW 41225	*Bruguiera gymnorrhiza*	South Africa	J.A. Osorio	2012	Osorio et al. (2016)	KP860852	KP860696	KP860774	KU587899
	CMW 41361	*Bruguiera gymnorrhiza*	South Africa	J.A. Osorio	2012	Osorio et al. (2016)	KP860850	KP860694	KP860772	KU587897
	CMW 41368	*Avicennia marina*	South Africa	J.A. Osorio	2012	Osorio et al. (2016)	KP860853	KP860697	KP860774	KU587900
***Neofusicoccum podocarpi*** W. Zhang & Crous	**CMW 35494**	*Podocarpus henkelii*	South Africa	J. Roux & M.L. Ndove	2008	Zhang et al. (2021)	MT587508	MT592223	MT592715	MT592412
	CMW 35499	*Podocarpus henkelii*	South Africa	J. Roux & M.L. Ndove	2008	Zhang et al. (2021)	MT587509	MT592224	MT592716	MT592413
***Neofusicoccum ribis*** (Slippers, Crous & M.J. Wingfield) Crous, Slippers & A.J.L. Phillips	CMW 7054	*Ribes rubrum*	USA	E. Stevens	2001	Slippers et al. (2004c)	AF241177	AY236879	AY236908	EU821960
	**CMW 7772**	*Ribes* sp.	USA	B. Slippers & G. Hudler	2000	Slippers et al. (2004c)	AY236935	AY236877	AY236906	EU821958
***Neofusicoccum stellenboschiana*** Tao Yang & Crous	CMW 13986	*Syzygium cordatum*	South Africa	D. Pavlic	2002	Pavlic et al. (2007)	DQ316085	MT592243	MT592735	MT592432
	CMW 41363	*Rhizophora mucronata*	South Africa	J.A. Osorio	2012	Osorio et al. (2016)	KP860829	KP860674	KP860752	MT592434
***Neofusicoccum terminaliae*** Begoude, Jolanda Roux & Slippers	CMW 26683	*Terminalia sericea*	South Africa	D. Begoude & J. Roux	2007	Yang et al. (2017)	GQ471804	GQ471782	KX465053	KX464046
	**CMW 26679**	* Terminalia sericea *	South Africa	D. Begoude & J. Roux	2007	Yang et al. (2017)	GQ471802	GQ471780	KX465052	KX464045
***Neofusicoccum umdonicola*** Pavlic, Slippers & M.J. Wingfield	CMW 13991	*Syzygium cordatum*	South Africa	D. Pavlic	2002	Pavlic et al. (2007)	DQ316075	MT592250	JF440836	MT592440
	CMW 14060	*Syzygium cordatum*	South Africa	D. Pavlic	2002	Pavlic et al. (2009)	EU821905	EU821875	EU821845	EU821935
	**CMW 14058**	*Syzygium cordatum*	South Africa	D. Pavlic	2002	Pavlic et al. (2009)	EU821904	EU821874	EU821844	EU821934
***Neofusicoccum ursorum*** Pavlic, Maleme, Slippers & M.J. Wingfield	CMW 23790	*Eucalyptus tree*	South Africa	H.M. Maleme	2006	Crous et al. (2013)	FJ752745	FJ752708	KX465057	–
	CMW 35471	*Podocarpus henkelii*	South Africa	E.M. Cruywagen & M.L. Ndove	2009	Zhang et al. (2021)	MT587527	MT592253	MT592745	MT592443
	CMW 35481	*Afrocarpus falcatus*	South Africa	E.M. Cruywagen & M.L. Ndove	2009	Zhang et al. (2021)	MT587526	MT592252	MT592744	MT592442
	**CMW 24480**	*Eucalyptus* tree	South Africa	H.M. Maleme	2006	Crous et al. (2013)	FJ752746	FJ752709	KX465056	KX464047
***Neofusicoccum variabile*** Marincowitz, Jami & M.J. Wingfield	CMW 37745	*Mimusops caffra*	South Africa	M.J. Wingfield	Unknown	Jami et al. (2018)	MH558608	–	MH569153	–
	**CMW 37739**	*Mimusops caffra*	South Africa	M.J. Wingfield	Unknown	Jami et al. (2018)	MH558610	MH576586	MH569155	–
***Neofusicoccum vitifusiforme*** (Van Niekerk & Crous) Crous, Slippers & A.J.L. Phillips	CMW 30605	*Acacia sieberiana*	South Africa	J. Roux	2008	Zhang et al. (2021)	MH863761	MT592256	MT592748	MT592446
	CMW 875	*Acacia mearnsii*	-	M.J. Morris	1987	Zhang et al. (2021)	MH863762	MT592257	MT592749	MT592447

Type strains are indicated in **bold** font.

**Table 5.** Results of the sporulation test for isolates in long-term storage on VMM.

Species name	Isolate	Isolation year	Days to initial pycnidia formation	Days to initial conidial discharge	Days to conidia harvest	Conidia mL^-1^
*Neofusicoccum andinum*	CMW 13446	2003	NP	NC	ND	ND
	**CMW 13455**	2003	NP	NC	ND	ND
*N. australe*	CMW 821	1986	NP	NC	ND	ND
	CMW 1133	1996	3	NC	ND	ND
	CMW 1187	1998	NP	NC	ND	ND
	**CMW 6837**	2000	NP	NC	ND	ND
	CMW 6838	2000	NP	NC	ND	ND
	CMW 6853	2000	NP	NC	ND	ND
	CMW 9073	1999	7	18	42	3.62 × 10^6^
*N. batangarum*	CMW 28320	2007	17	NC	ND	ND
	**CMW 28363**	2007	NP	NC	ND	ND
*N. cordaticola*	**CMW 13992**	2002	NP	NC	ND	ND
	CMW 14056	2002	7	NC	ND	ND
*N. cryptoaustrale*	**CMW 23785**	2006	7	30	42	0
*N. dianense*	CMW 54123	2018	19	30	42	1.5 × 10^5^
	CMW 54139	2018	NP	NC	ND	ND
	CMW 54189	2018	10	30	42	1.75 × 10^5^
*N. eucalypticola*	CMW 6217	2000	10	NC	ND	ND
	CMW 6229	2000	10	NC	ND	ND
	**CMW 6539**	2000	19	30	42	1.24 × 10^7^
	CMW 6543	2000	19	30	42	1.27 × 10^6^
*N. eucalyptorum*	CMW 6233	2000	19	30	42	0
	**CMW 10125**	2001	19	30	42	6.75 × 10^5^
*N. kwambonambiense*	**CMW 14023**	2002	NP	NC	ND	ND
	CMW 14140	2003	NP	NC	ND	ND
*N. lumnitzerae*	CMW 41228	2012	4	32	42	6.75 × 10^5^
	CMW 41613	2011	7	NC	ND	ND
	**CMW 41469**	2012	7	NC	ND	ND
*N. luteum*	CMW 14071	2002	NP	NC	ND	ND
*N. mangiferae*	CMW 7797	1988	NP	NC	ND	ND
	**CMW 7024**	1988	NP	NC	ND	ND
*N. mangroviorum*	**CMW 41365**	2012	7	NC	ND	ND
	CMW 42355	2011	NP	NC	ND	ND
	CMW 42481	2011	7	NC	ND	ND
*N. microconidium*	CMW 13998	2002	38	NC	ND	ND
*N. ningerense*	CMW 54121	2018	7	NC	ND	ND
*N. ningerense*	CMW 54144	2018	7	21	42	1.5 × 10^5^
	CMW 54212	Unknown	10	24	42	5 × 10^4^
*N. occulatum*	CMW 54187	2018	4	24	42	0
*N. pandanicola*	CMW 14029	2002	14	NC	ND	ND
	CMW 14087	2002	18	32	42	5 × 10^4^
*N. parvum*	CMW 9071	2000	7	NC	ND	ND
	CMW 9080	2005	NP	NC	ND	ND
	**CMW 9081**	2005	4	NC	ND	ND
	CMW 13350	2002	NP	NC	ND	ND
	CMW 41225	2012	4	NC	ND	ND
	CMW 41361	2012	NP	NC	ND	ND
	CMW 41368	2012	NP	NC	ND	ND
*N. podocarpi*	**CMW 35494**	2008	NP	NC	ND	ND
	CMW 35499	2008	10	NC	ND	ND
*N. ribis*	CMW 7054	2001	24	NC	ND	ND
	**CMW 7772**	2000	NP	NC	ND	ND
*N. stellenboschiana*	CMW 13986	2002	NP	NC	ND	ND
	CMW 41363	2012	NP	NC	ND	ND
*N. terminaliae*	CMW 26683	2007	NP	NC	ND	ND
	**CMW 26679**	2007	NP	NC	ND	ND
*N. umdonicola*	CMW 13991	2002	NP	NC	ND	ND
	CMW 14060	2002	7	NC	ND	ND
	**CMW 14058**	2002	NP	NC	ND	ND
*N. ursorum*	CMW 23790	2006	NP	NC	ND	ND
	CMW 35471	2009	14	NC	ND	ND
	CMW 35481	2009	NP	NC	ND	ND
	**CMW 24480**	2006	NP	NC	ND	ND
*N. variabile*	CMW 37745	Unknown	10	24	42	5 × 10^4^
	**CMW 37739**	Unknown	7	NC	ND	ND
*N. vitifusiforme*	CMW 30605	2008	7	24	42	5 × 10^4^
	CMW 875	1987	NP	NC	ND	ND

**NP**, no pycnidia formation; **NC**, no conidia discharge; **ND**, no data.

Among all *Neofusicoccum* species evaluated (Table 5), the highest conidial concentration was recorded for *N. eucalypticola* isolate CMW 6539, yielding 1.24 × 10^7^ conidia mL^-1^. In contrast, isolate CMW 6543 of the same species produced substantially fewer conidia (1.27 × 10^6^ conidia mL^-1^). *Neofusicoccum australe* isolate CMW 9073 produced 3.62 × 10^6^ conidia mL^-1^, followed by *N. eucalyptorum* isolate CMW 10125 and *N. lumnitzerae* isolate CMW 41228, both yielding 6.75 × 10^5^ conidia mL^-1^. *Neofusicoccum dianense* isolates CMW 54189 and CMW 54123 produced 1.75 × 10^5^ and 150,000 conidia mL^-1^, respectively. The lowest concentrations were observed in *N. ningerense* (CMW 54212), *N. pandanicola* (CMW 14087), *N. variabile* (CMW 37745), and *N. vitifusiforme* (CMW 30605), all with 5.0 × 10^4^ conidia mL^-1^, although isolate CMW 54144 of *N. ningerense* produced 1.5 × 10^5^ conidia mL^-1^.

**Figure 7.**
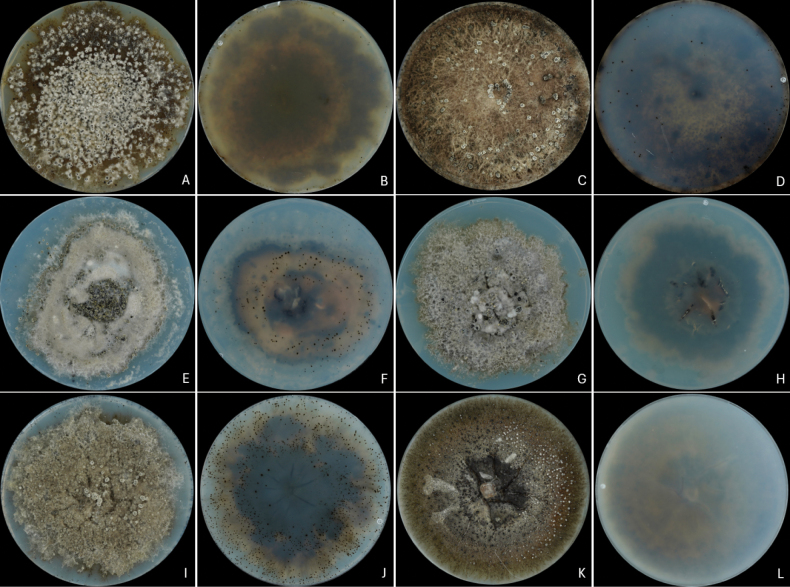
Sporulation of *Neofusicoccum* strains after long-term preservation and subsequent incubation on VMM for 28 days. **A, B** Front and reverse sides of *N. australe* CMW 9073; **C, D***N. cryptoaustrale* CMW 23785; **E, F***N. eucalypticola* CMW 6543; **G, H***N. eucalyptorum* CMW 10125; **I, J***N. ningerense* CMW 54212; **K, L***N. vitifusiforme* CMW 30605.

No association was detected between sporulation on VMM and the geographic origin of the isolates or their host of isolation (Fig. 7). In contrast, a relationship was observed with culture age, defined by date of isolation. Isolates obtained during the 1980s consistently failed to produce pycnidia or conidia under the tested conditions. By comparison, a few isolates from the late 1990s, such as *N. australe* isolates CMW 1133 (1996) and CMW 9073 (1999), formed pycnidia, although conidial production was limited. Isolates from 2000–2007 displayed greater variability, with some strains yielding high conidial concentrations (e.g., *N. eucalypticola* isolates CMW 6543 and CMW 6539, both isolated in 2000, producing 1.27 × 10^6^ and 1.24 × 10^7^ conidia mL^-1^, respectively), while others from the same period showed no sporulation. More recent isolates (2010–2018) generally displayed higher sporulation frequencies, exemplified by *N. dianense* isolates CMW 54123 and CMW 54189 (2018), producing 1.5 × 10^5^ and 1.75 × 10^5^ conidia mL^-1^ respectively, and *N. ningerense* isolate CMW 54144 (2018), which reached 1.5 × 10^5^ conidia mL^-1^ in some cases.

## Discussion

In this study, we extend the work of Oostlander et al. (2023) by testing and refining their sporulation protocol for *D. africana*, *D. mutila*, *D. seriata*, *L. brasiliensis*, and *L. theobromae*, as well as 26 species of *Neofusicoccum*. Our findings demonstrate that VMM, under constant white light at 25 °C, effectively promotes the formation of pycnidia and the discharge of conidia in a wide array of taxa, but with notable differences between species and isolates. Such inter- and intraspecific variation is not unexpected in the *Botryosphaeriaceae* and likely reflects a combination of biological and culture-related factors. Species-specific developmental regulation and nutritional requirements can influence both the timing of pycnidial initiation and the efficiency of conidial discharge, which is consistent with the contrasting sporulation responses observed across taxa under different sucrose concentrations in our assays. In addition, isolate-level variation may reflect differences in physiological status linked to culture history (e.g. number of transfers, growth morphology, and the extent of aerial mycelium), which can affect pycnidial maturation and cirri production even under otherwise standardised conditions. Moreover, culture age and long-term storage history can reduce reproductive competence, as reflected by the progressive loss of sporulation capacity observed in older strains in our work. This study therefore represents a significant advancement in the development of standardised and reproducible methodologies for inducing sporulation in members of the *Botryosphaeriaceae*.

The protocol described in this study using VMM demonstrated rapid, reproducible, and substrate-independent sporulation. Pycnidia development began within three days for *D. seriata*, four days for *N. cryptoaustrale* and *N. luteum*, and five days for *L. brasiliensis*, *L. theobromae*, *N. australe*, *N. parvum*, and *N. stellenboschiana*. The quantity of conidia produced exceeded those reported in previous studies. For instance, *N. luteum* reached a mean concentration of 1.5 × 10^7^ conidia mL^-1^, more than 600 times higher than the highest value reported by Amponsah et al. (2008). Notably, both studies identified *N. luteum* as the most prolific species in terms of conidia production. The VMM protocol not only excels in speed and yield but also in operational simplicity. It eliminates the need for plant tissue substrates and photoperiod control. Cultures maintained under constant white light at 25 °C showed consistent pycnidia formation in most species. Moreover, the release of conidia in cirri by the majority of species further simplified harvesting, which was achieved by flooding plates with 0.01% Tween 20, followed by gentle agitation, centrifugation, and final resuspension. Unlike traditional methods, there was no need for manual disruption of pycnidia.

When comparing our VMM-based results with those of Oostlander et al. (2023) for *D. sapinea*, it is notable that some species and strains using the protocol described here produced even higher sporulation levels. Oostlander et al. (2023) reported a maximum of 1.7 × 10^6^ conidia mL^-1^ per plate (5.5 cm diameter). In our assays, using two 6.0 cm plates per isolate, *D. seriata* produced 1.3 × 10^6^ conidia mL^-1^, comparable to the values reported by Oostlander et al. (2023). However, significantly higher yields were recorded for *L. brasiliensis* (8.7 × 10^6^ and 7.12 × 10^6^ conidia mL^-1^), *N. parvum* (2.31 × 10^6^ conidia mL^-1^), *N. stellenboschiana* (2.0 × 10^6^ conidia mL^-1^), and particularly *N. luteum* (1.5 × 10^7^ conidia mL^-1^). These data confirm the broad applicability of VMM beyond *Diplodia*, extending its efficacy to support spore-based studies across the *Botryosphaeriaceae*.

In our study, continuous white light at 25 °C favoured pycnidia formation and conidia discharge. Light conditions have long been recognised as influential in fungal development (Smith and Fergus 1971). Although previous research, such as Úrbez-Torres and Gubler (2009), demonstrated that alternating light and dark periods promoted sporulation in some species, the constant white light conditions initially established by Oostlander et al. (2023) proved effective for most taxa tested here. Nevertheless, future studies should evaluate whether varying light regimes (e.g. white light-dark cycles, variation in light wavelength, or near-UV exposure) could further enhance sporulation outcomes across additional species or isolates using VMM.

Sucrose concentration in VMM had a substantial impact on pycnidia development and conidia discharge, underscoring the critical role of carbon availability in regulating sporulation efficiency in the *Botryosphaeriaceae*. Although all tested species formed pycnidia across a range of sucrose levels, both the timing and quantity of conidia released varied considerably. Optimal sporulation was observed at sucrose concentrations of 1.5%–1% for *L. brasiliensis*, *L. theobromae*, *N. luteum*, *N. parvum*, and *N. stellenboschiana*, suggesting that balanced carbon availability supports not only structural development but also the metabolic processes required for active conidia release. In contrast, low or absent sucrose levels delayed pycnidia formation and complicated sporulation in species such as *D. mutila* and *N. cryptoaustrale*. Notably, *D. africana* formed pycnidia at all sucrose concentrations, and conidial discharge occurred at 1.5%, 1%, and 0%; however, conidia were microscopically observed only at 0% sucrose, indicating delayed and markedly reduced sporulation compared with other species. In contrast, *D. seriata* showed optimal sporulation at 0.5% and 0.25% sucrose. These patterns indicate that sucrose functions not only as a nutrient but also potentially as a regulatory cue in reproductive development. Precise adjustment of sucrose concentration is therefore essential for optimising sporulation protocols.

Geographic origin and host species did not influence sporulation capacity in this study, whereas a progressive loss of sporulation capacity was associated with increasing culture age. Strains collected from Australia, Indonesia, Spain, and South Africa all demonstrated the ability to produce pycnidia and conidia under standardised conditions. Isolates obtained by the Instituto Canario de Investigaciones Agrarias (Spain) between 2021 and 2024 showed robust sporulation, whereas older strains from the CMW collection (isolated between 1986 and 2018 and maintained on MYA at 4 °C since isolation) exhibited reduced capacity, with some failing to sporulate entirely. This observation is consistent with previous findings by Baskarathevan et al. (2009) and Tariq et al. (2015), suggesting that long-term storage or repeated subculturing may compromise reproductive viability. Nonetheless, the presence of older isolates with high conidial yields (e.g. *N. australe* CMW 9073, producing 3.62 × 10^6^ conidia mL^-1^ after storage since 1999) indicates that additional factors such as species identity, storage conditions, or the number of prior subcultures also influence sporulation capacity. Isolates and conidial suspensions obtained using this protocol can be cryopreserved in 40% glycerol at −80 °C, as described by Oostlander et al. (2024). Further studies are required to determine optimal preservation conditions for individual species and to assess whether cryopreservation under these or alternative conditions affects strain viability and subsequent sporulation.

Species-level sporulation patterns revealed marked differences across genera. *Diplodia seriata* consistently exhibited the fastest and most abundant sporulation across several sucrose concentrations, whereas *D. africana* and *D. mutila* showed poor or no conidial discharge, suggesting species-specific limitations or unidentified sporulation triggers. *Lasiodiplodia brasiliensis* and *L. theobromae* sporulated efficiently under all conditions tested. In *Neofusicoccum*, sporulation behaviour varied widely both among species and among isolates. *Neofusicoccum luteum* was the most efficient species at a sucrose concentration of 1.5%. In contrast, *N. australe* and *N. cryptoaustrale* sporulated better at lower sucrose concentrations, whereas *N. parvum* and *N. stellenboschiana* showed delayed and inconsistent sporulation, with lower conidial yields and some isolates failing to form pycnidia.

Several media have previously been used to induce sporulation in *Botryosphaeriaceae* species, often supplemented with plant material. These include 2% MEA, 2% PDA, Oatmeal Agar (OA) (Crous et al. 2019), 2% water agar (Phillips et al. 2013), and Prune Agar (PA) (Amponsah et al. 2008). A limitation of these media is their fixed composition, which restricts precise manipulation of individual components for investigating the influence of specific chemical variables on sporulation. In contrast, VMM allows precise modification of each constituent component, enabling the formulation to be tailored to the physiological requirements of target species. Among its components, sucrose serves as a key carbon source, and its concentration was shown here to strongly influence sporulation efficiency. Oostlander et al. (2023) conducted a side-by-side evaluation of several commonly used culture media (CD, LNA, OA, and PDA) alongside VMM under controlled incubation conditions and observed pycnidia formation and harvestable spores on VMM (and VMM supplemented with pine needles), but not on CD, LNA, OA, or PDA for *D. sapinea*. Based on this evidence, and because the primary objective of the present study was to establish a single standardised protocol applicable across a large and taxonomically diverse isolate set, we focused on VMM and optimisation of its key parameters rather than including parallel medium controls for each isolate. Nevertheless, systematic cross-medium comparisons across multiple taxa remain an important direction for future research.

Plant materials, particularly pine needles but also twigs from various woody plants, are commonly used as substrates to stimulate sporulation in the *Botryosphaeriaceae*. Similar approaches are applied to other fungal taxa, such as leaves of *Rhododendron pulchrum* for *Guignardia endophyllicola* (*Phyllosticta capitalensis*) (Okane et al. 2001), *Dianthus caryophyllus* leaves for *Fusarium* species (Fisher et al. 1982) and *Pestalotiopsis* species (Liu et al. 2010), or *Gardenia jasminoides* and *Hydrangea macrophylla* leaves to induce perithecia and ascospores in *Glomerella singulata*, *Mycosphaerella allicina*, *Metasphaeria* sp., *Guignardia* sp., and *Pleospora herbarum* (Furukawa and Kishi 2002). However, plant materials are not always readily available, and their chemical composition can vary depending on host species, geographic origin, and physiological state. This variability introduces inconsistency into sporulation studies, whereas the use of VMM provides compositional consistency that ensures reproducibility across experiments and laboratories. Moreover, while plant substrates restrict pycnidia development to their surfaces, VMM enables pycnidia formation across the entire Petri dish.

## Conclusion

Collectively, these findings demonstrate that VMM offers a defined, reproducible, and technically simple framework for inducing sporulation in the *Botryosphaeriaceae* under the incubation conditions tested here. Its defined composition, high reproducibility, technical simplicity, and substantial conidial yields make it a valuable and versatile tool for both mycological and plant pathology research. In a fungal family characterised by pronounced interspecific and intraspecific variability in sporulation behaviour, the establishment of a medium applicable to species from three genera represents a significant methodological advancement. This study provides a robust quantitative and procedural framework that bridges laboratory experimentation and ecological or applied contexts, supporting cross-laboratory reproducibility and enabling more controlled studies. However, further research is required, including systematic cross-medium comparisons under identical incubation conditions, to optimise VMM formulations for specific taxa, uncover the molecular and physiological mechanisms underpinning sporulation, and integrate morphological and molecular data to enhance taxonomic resolution.

## Additional information

### Use of AI

No use of AI was reported.

### Adherence to national and international regulations

All the fungal strains used in this study have been legally obtained, respecting the Convention on Biological Diversity (Rio Convention).

### Author ORCIDs

David Hernández-Hernández https://orcid.org/0000-0002-7017-7296

Felipe Siverio de la Rosahttps://orcid.org/0000-0002-8886-414X

Christiaan Grobler https://orcid.org/0000-0001-8497-765X

Bernard Slippers https://orcid.org/0000-0003-1491-3858

### Data availability

All sequence data obtained or used in this study are available in GenBank (https://www.ncbi.nlm.nih.gov/genbank/).

## Supplementary materials

Supplementary material 1 Additional tables and figures Data type zip Explanation note **table SS1**. Collection data and GenBank accession numbers of 123 *Botryosphaeriaceae* isolates from the Canary Islands. **table SS2**. Sporulation data of fungal isolates collected in the Canary Islands, Spain. **table SS3**. Collection data and GenBank accession numbers for isolates included in the phylogenetic analyses of the genus *Diplodia*. **table SS4**. Collection data and GenBank accession numbers for isolates included in the phylogenetic analyses of the genus *Lasiodiplodia*. **table SS5**. Collection data and GenBank accession numbers for isolates included in the phylogenetic analyses of the genus *Neofusicoccum*. **fig. S1**. Phylogenetic tree of *Diplodia* resulting from a Bayesian analysis of the combined ITS, *tef*1-α, and *tub*2 sequence alignment. **fig. S2**. Phylogenetic tree of *Lasiodiplodia* resulting from a Bayesian analysis of the combined ITS, *tef*1-α, *tub*2, and *rpb*2 sequence alignment. **fig. S3**. Phylogenetic tree of *Neofusicoccum* resulting from a Bayesian analysis of the combined ITS, *tef*1-α, *tub*2, and *rpb*2 sequence alignment. https://binary.pensoft.net/file/1534006This dataset is made available under the Open Database License (http://opendatacommons.org/licenses/odbl/1.0/). The Open Database License (ODbL) is a license agreement intended to allow users to freely share, modify, and use this Dataset while maintaining this same freedom for others, provided that the original source and author(s) are credited. David Hernández-Hernández, Felipe Siverio de la Rosa, Christiaan Grobler, Bernard Slippers
